# Putting CRISPR-Cas system in action: a golden window for efficient and precise genome editing for crop improvement

**DOI:** 10.1080/21645698.2023.2219111

**Published:** 2023-06-08

**Authors:** Arooj Tariq, Muntazir Mushtaq, Huwaida Yaqoob, Basharat Ahmad Bhat, Sajad Majeed Zargar, Ali Raza, Sajad Ali, Sidra Charagh, Muhammad Salman Mubarik, Qamar U Zaman, PV Vara Prasad, Rakeeb Ahmad Mir

**Affiliations:** a Department of Biotechnology, School of Biosciences and Biotechnology, BGSB University, Rajouri, J&K, India; b MS Swaminathan School of Agriculture, Shoolini University of Biotechnology and Management Sciences, Bajhol, Solan, India; c Department of Bioresources, School of Biological Sciences, University of Kashmir, Srinagar, J&Kr, India; d Proteomics Laboratory, Division of Plant Biotechnology, (SKUAST-K), Shalimar, Kashmir, India; e College of Agriculture, Fujian Agriculture and Forestry University (FAFU), Fuzhou, China; f Department of Biotechnology, Yeungnam University, Gyeongsan, South Korea; g State Key Laboratory of Rice Biology, China National Rice Research Institute, Chinese Academy of Agricultural Sciences (CAAS), Hangzhou, Zhejiang, China; h Department of Biotechnology, University of Narowal, Narowal, Pakistan; i Yazhou Bay Seed Laboratory, Sanya Nanfan Research Institute of Hainan University, Sanya, China; j College of Tropical Crops, Hainan University, Haikou, China; k Department of Agronomy, Kansas State University, Manhattan, KS, USA; l Department of Biotechnology, School of Life Sciences, Central University of Kashmir, Ganderbal, J&K, India

**Keywords:** breeding, CRISPR/Cas system, crop improvement, future crops, GE technologies, mutagenesis, wild crops

## Abstract

The daunting task of feeding an ever-growing population is an immense challenge for the contemporary scientific community, especially in view of the rapidly changing climate throughout the world. Amidst these threatening crises, we witness rapid development in genome editing (GE) technologies, revolutionizing the field of applied genomics and molecular breeding. Various GE tools have been developed during the last two decades, but the CRISPR/Cas system has most recently made a significant impact on crop improvement. The major breakthroughs of this versatile toolbox are genomic modifications like single base-substitutions, multiplex GE, gene regulation, screening mutagenesis, and enhancing the breeding of wild crop plants. Previously, this toolbox was used to modify genes related to significant traits such as biotic/abiotic resistance/tolerance, post-harvest traits, nutritional regulation, and to address self-incompatibility analysis-related challenges. In the present review, we have demonstrated the functional dynamics of CRISPR-based GE and its applicability in targeting genes to accomplish novel editing of crops. The compiled knowledge will provide a solid foundation for highlighting the primary source for applying CRISPR/Cas as a toolbox for enhancing crops, to achieve food and nutritional security.

## 1. Introduction

The need for technical advancements to feed the growing world population is inevitable, and plant scientists constantly deal with this challenge. The outstanding contribution of the Green revolution and new technical advances in plant breeding efforts are underway to overcome the challenge to achieve global food security under changing climate conditions.^1–6^ Since ancient times, humans have improved crop plants by transferring novel traits from crossable relatives. The sole motive of this practice was to transfer desirable gene variants even though the process is time-consuming as it takes several years to transfer alleles of interest to desirable genotype. With the advancements, breeders learned to use chemical mutagens or radiations to generate mutants with the desired modifications in plants.^7^ The yield and quality of crops have exceptionally increased by using spontaneous and induced mutations and transgenic methods.^8–16^ Despite the availability of appropriate germplasm for crop improvement, these mutations have proven to be time-consuming and labor-intensive due to their low frequency and unpredictability.^7^ The last decade witnessed a revolution in crop improvements with the introduction of various genome-editing tools.^17,18^ Previous reports have demonstrated that the effective strategies for targeted mutagenesis at the target loci can be customized using sequence-specific nucleases (SSNs), which generate double-strand breaks (DSBs) in DNA.^19,20^

The major drawback of transgenic crops lies in the tedious risk assessment and cost that may lead to less production and the application of traditional transgenic approaches. Further integration of foreign genes through illegitimate recombination may lead to sequence changes, gene disruption, random transgene integration, and production of irrelevant mutant proteins.^21,22^ In contrast, GE tools such as, CRISPR-Cas9-based GE, are the most amendable tools for the desirable editing of genomes in diverse range of plant species. Qualities such as, high efficacy, low off-target effects, simplicity in operation, cost effectiveness, and multiplex gene editing make them the most dominantly employed tools for plant modifications. In comparison to traditional transgenic approaches, the CRISPR/Cas9 system is incommensurable in the development of sustainable agriculture.^15,23–27^

Advanced GE tools such as zinc-finger nucleases (ZFNs), transcription activator-like effector nucleases (TALENs), and CRISPR-Cas systems are employed to accomplish mutagenic effects in an organism, for inducing desirable changes in a predictable manner.^15,17,28–30^ These techniques generate double-stranded breaks (DSBs) in the target DNA and induce deletions or insertions of nucleotides by the endogenous repair system via non-homologous end joining (NHEJ) or precise gene insertion or gene replacement by homologous recombination (HR), resulting in a loss-of-function or gain-of-function of the target genes.^31^ For instance, to attain these changes in the target genome, earlier, scientists applied GE tools such as, ZFN and TALEN; both rely on artificial restriction enzymes that exert cleavage functions by the combination of the DNA- binding domains (DBDs) of ZFN and TALENs with the cleavage domains of Fok1.^32–34^ Thus, through the protein-DNA interactions, both ZFN and TALEN identify their targeted sequences. Even though effective mutagenesis of ZFN and TALEN have been reported in many plant species, the difficult and prolonged DNA assembly procedure of ZFN/TALEN DNA- binding component has been challenging for scientists, consequently hindering their effective applications.^35,36^

In recent years, the advancements and developments in engineered CRISPR-Cas9 system has revolutionized targeted GE in plants and animals as it can recognize the target sites only via a single-guide RNA (sgRNA), unlike ZFN and TALEN.^37,38^ With the introduction of the CRISPR-toolbox, scientists are now better able to comprehend and read genomic structure better than ever before as well as edit it with previously unreachable precision.^39^ The construction of the Cas9 RNA-guided engineered nuclease (RGEN) system is far more simple and easy, versatile, specific, and faster than assembling numerous ZFNs or TALENs for GE.^40,41^ Currently, the CRISPR/Cas9 system has emerged as a powerful tool for GE in a large variety of plant species, right from the model plant *Arabidopsis* and rice to other crop plants such as tetraploid potato, maize, hexaploid bread wheat, allotetraploid cotton, and tetraploid durum wheat.^35,42–46^ In addition, the CRISPR/Cas9 system was employed to target several homologous sites in *Brassica napus* genes, such as *BnaRGA, BnaDA2, BnALC*, *BnJAG*, *BnSHP*, *BnCLV*, and *BnaFUL*.^47–50^ Even though employed in a wide range of plants, it still has not developed an easy and quick tool to generate first-generation mutated plants, which are important to gene functionalization and screening of mutations in a later generation.

**Figure 1.**
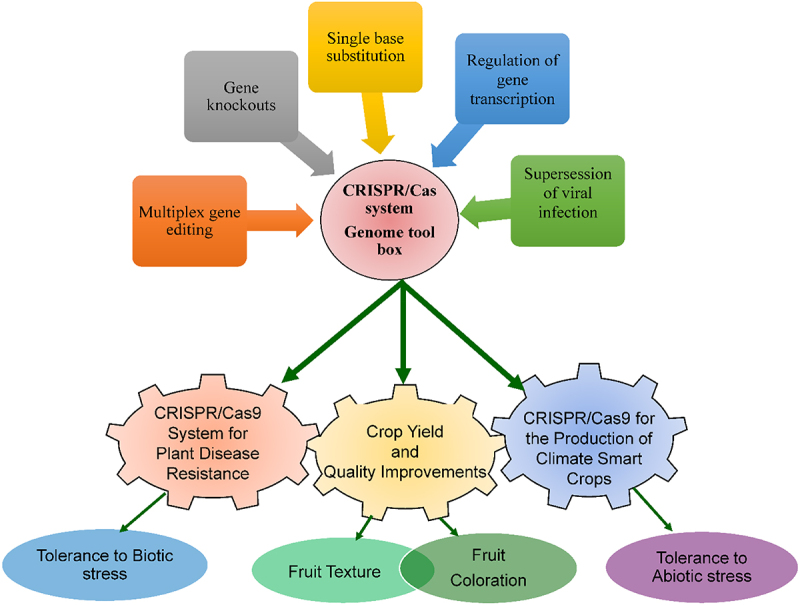
Illustration for developing superior and elite crop plants resilient to biotic and abiotic stress factors, and enhancing the quality of crops by using CRISPR/Cas systems through single base substitution, multiplex gene editing, gene knockout, regulation of gene transcription, and enhancing and screening mutagenesis.

Overall, the engineered CRISPR/Cas9 system involves two components, Cas9 protein expression cassettes and sgRNA. Several modifications mediated by CRISPR/Cas system include single base substitution, multiplex gene editing, regulation of gene transcription, and enhancing, screening mutagenesis, and enhancing the breeding of wild crop plants to enhance resilience to biotic as well abiotic stresses (Fig. 1).^27,38,51^ The current review is aimed at comprehensively demonstrating the versatility of the CRISPR/Cas-based GE systems to accomplish efficient crop improvement for desirable traits to enhance the productivity, stress tolerance, and nutritional profile of crop plants. The following sections in this review will highlight the various mechanisms that CRISPR-based GE techniques work and the variety of approaches that they can be used to change plant genomes for the benefit of humanity’s existence. In addition, we have also critically mentioned the biosafety concerns over the dissemination of crops engineered through CRISPR-based tools.

## 2. Frontline Approach to Crop Improvement-CRISPR-Cas System-Construction and Mechanistic Approach

The development and advancements in the CRISPR/Cas GE tools have allowed them to emerge as crucial tools in biological research to understand the function of genes and in crop improvement.^52^ The different classes of GE agents that are presently available for editing the genome include – nucleases, base editors, and prime editors. Each tool has its own simplicity, versatility, and limitations, and the major efforts and improvements in the editing capabilities of the tools have broadened and extended their targeting as well as enhanced specific editing abilities. The suitable class of editing agents used in experimental systems depends on the type of editing that is desired. The common editings yielded by CRISPR/Cas include (i) point mutations, that is, conversion of DNA base pairs, (ii) insertion of DNA base pairs, (iii) deletion of DNA base pairs or (iv) a combination of the above changes (including replacement of DNA base pairs).

### 2.1. CRISPR/Cas Nucleases: A Technical Overview

**Figure 2.**
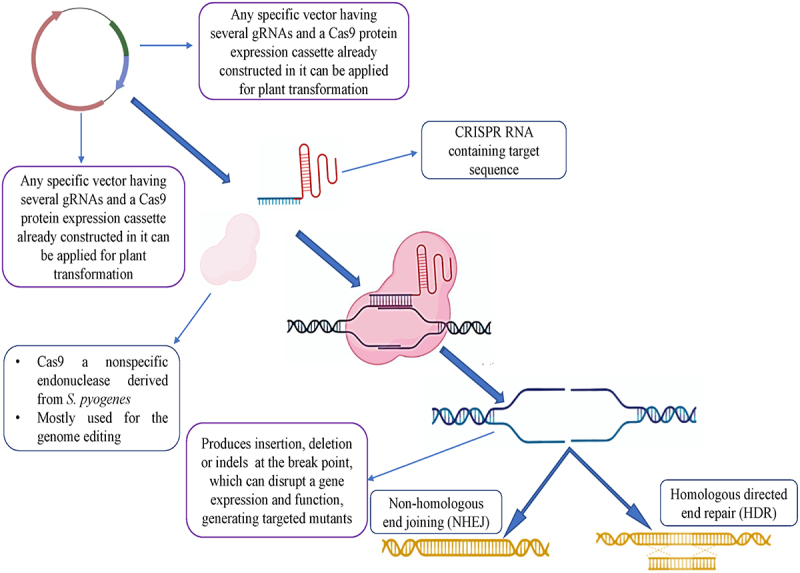
Schematics show the mechanistic workflow of the CRISPR/Cas system and its precise catalysis by inducing editing through NHEJ or HDR, employed to improve crops.

Naturally, by binding, CRISPR-Cas systems are used by bacteria and archaea as a part of their adaptive immunity to protect the host from foreign pathogens and to cleave foreign nucleic acids.^53^ The mechanistic chemistry of CRISPR/Cas systems in practice is reprogrammed readily in order to target specific genomic sites by simply using the spacer sequences within a guide RNA molecule and by providing the similar target DNA protospacer sequence that is placed adjacent to a protospacer-adjacent motif (PAM), generating nonhomologous end joining (NHEJ) or homology-directed repair (HDR) (Fig. 2).^29,54,55^ Furthermore, there are two main groups of naturally occurring CRISPR/Cas immune systems, *viz*., class-I and class-II. As far as class-1 is concerned, it uses the multiprotein complexes to cleave nucleic acids and is very rarely used for editing genomes. Whereas, class-II employs only one protein effector domain to cleave the nucleic acids. Due to this advantage, class-II is the most widely used CRISPR tool for biological research and translation applications.^56,57^ Class-II has been further divided into the three types, II, V, and VI; each of these types functions by employing different sets of Cas proteins. Among these, the Cas 9 variants from type – II and Cas 12 variants from type-V have the RNA-guided DNA endonuclease activity whereas Cas 13 of type VI possesses the targeting activity of RNA and the cleavage activity.

#### 2.1.1. Cas9 Nucleases

The Cas9 protein, RNA-guided endonucleases, forms a part of the type-II CRISPR systems can generate DSBs in the target DNA.^58^ The Cas9 nucleases and the CRISPR RNAs (crRNA) form the ribonucleoprotein complex to mediate its action on the target DNA sequence.^59^ The Cas-9 GE uses the synthetic sgRNAs, a fusion product of crRNA and trans-activating CRISPR RNA (tracrRNA) molecules.^29^ The Cas9 nuclease primarily makes the blunt-ended DSBs at the 3 bp upstream of the PAM sequences such as, 5′-NNGRR(N)-3´^60^ and, in some cases, the Cas9, where alternative cutting patterns have been observed.^61,62^ The Cas9-guide RNA ribonucleoprotein complex recognizes the similar PAM sequences and induces unwinding of the target dsDNA to form RNA-DNA heteroduplex.^58,63^ Further, the guide RNA spacer displaces DNA at the nontarget sites, leading to the formation of the single-stranded “R-loop,” which is available to other molecules for interaction.^64–66^ Subsequently, the formation of the R-loop and the conformation of Cas9 changes result in the activation of its nuclease domains.^29,64,66,67^ The mismatches formed between the guide RNA spacer and the target strand hinders the conformational changes in the Cas9 protein complex, thus restricting its activation.^68,69^ Lastly, after the nuclease activation, two distinct nuclease domains of Cas9 hydrolyze the phosphodiester backbone of DNA. Subsequently, RuvC nuclease domain cleaves the PAM-containing nontarget DNA strand and the HNH nuclease domain induces cleavage in the guide RNA-bound target DNA strand.^70,71^ Moreover, it is reported that the mutation induced in either of nuclease domains generates a Cas9 nickase, which is capable of cleaving only one DNA strand. On the other hand, inactivation of both the nuclease domains generates a catalytically dead Cas9 (dCas9).^23,72,73^ Ever since the reports of programmed DNA cleavage by Cas9 nuclease from *Streptococcus pyogenes* (SpCas9) in vitro^70^ and in mammalian cells,^23,74–76^ the Cas9 variants discovered and used for the GE orthologs are derived from microorganisms such as, *Staphylococcus aureus*,^77^ *Streptococcus thermophiles*,^23,75,78^ *Neisseria meningitidis*,^78–80^ *Campylobacter jejuni*,^81^ and other reported organisms.^82^

#### 2.1.2. Cas12 Nucleases

The Cas12 nucleases include numerous variations from the type-V CRISPR/Cas system.^83^ They attain a single RuvC-like domain that induces cleavage in the target DNA in both strands. The first Cas12 nuclease widely used for GE applications is Cas12a, also known as Cpf1.^84^ Cas12a facilitates multiplexed gene editing as it can use a dedicated RNase domain to process its single crRNAs.^85^ Several Cas12 variants, including Cas12a, follow target site recognition and subsequent activation as well as cleavage of ssDNA or RNA.^86,87^ Although most Cas12 variants target and cleave DNA, other Cas12 effectors, such as Cas12g, are RNA-guided RNA-cleaving enzymes that exhibit both collateral RNase and DNase activity, upon activation.^86^ Different from Cas9, the Cas12a identifies and recognizes the T-rich PAM sequence, resulting in the maturation of their own guide RNA and generates a staggered 5′ and 3′ ends breaks in PAM distal dsDNA.^84^ For instance, resistance to high temperature was obtained by using Cas12b for editing *GhCLA* gene in the genome of cotton (*Gossypium hirsutum*).^88^ Similarly, the LbCpf1 genome editing tool was used to induce DSBs for replacing wild type ALS with mutated ALS for obtaining herbicide resistance in japonica rice (*Oryza sp*.).^89^ In rice, LbCas12a was used to mediate site-specific mutation in the Xa13 gene to develop resistance to bacterial leaf blight.^90^ The abovementioned studies clearly indicate the versatile nature of the Cas12-based GE systems have great potential in improving the resilience of crop plants.

### 2.2. The Overview of Genome Editing (GE) of Specific Genomic Regions

#### 2.2.1. Base Editors-A Narrow Window for CRISPR/Cas Based GE

**Figure 3.**
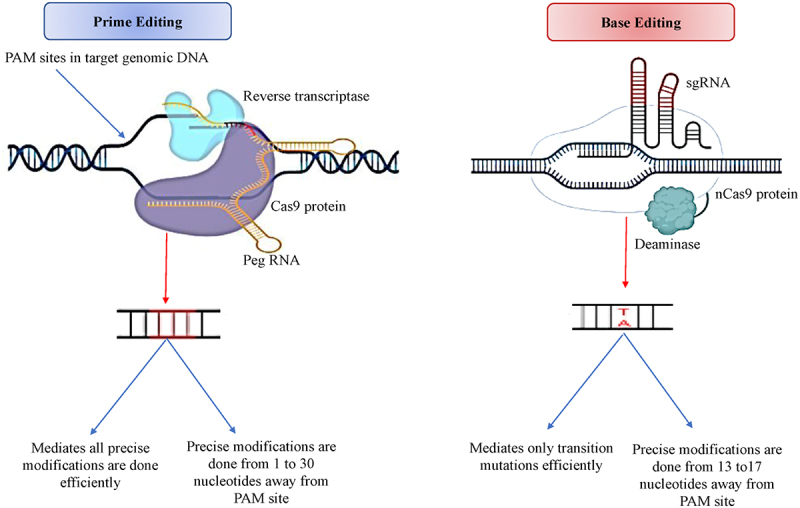
Ways toward precise editing: The prime editing mechanism involves the formation of a complex that comprises pegRNA to yield single-strand nick at a precise position in the target DNA PAM sequences, followed by the polymerization of newly edited DNA by reverse transcriptase by using pegRNA as a template. Subsequently, the sgRNA is employed to form the nick on the non-modified DNA strand to initiate repair-based insertion of editing in both the DNA strands. Base editied nCas9 is the preferred nuclease employed in dbase editing to generate single-stranded nick in the target DNA stranded, aided by gRNA using base deaminase to accomplish a single or more preferred nucleotide base exchanges.

Improved crop cultivars can be developed through CRISPR/Cas9-mediated mutagenesis to address growing food needs. In particular, single base editing through CRISPR/Cas9 could result in elite trait variants that hasten crop improvement programs. The base editing technology is an alternative genome-diting tool that generates point mutations precisely without making DSBs in the target region of genomes (Fig. 3). As an alternative, the Cas9 nickase (nCas9) is fused with the base editor and the guide RNA to target a specific DNA sequence. To date, two primary base editing tools have been reported, viz., cytosine base editors (CBEs) and adenine base editors (ABE).^91,92^ For instance, the nCas9, a CBEs causes a mutation in D10A, resulting in the inactivation of the RuvC domain. A wide range of crop genomes such as, rice, maize (*Zea mays*), wheat (*Triticum aestivum*), and potato (*Solanum tuberosum*) has been precisely edited by the CBE-mediated base editing technology to obtain the desired results.^91,93^

Adenine base editing is another potential CRISPR system that converts A-T to G-C. The adenosine deaminase activity of the enzyme helps in deaminating adenosine to produce inosine, which can then be paired with cytidine. The deaminated nucleotide is identified as guanine by the DNA polymerase during the process of DNA replication and repair mechanism.^94^ The ABE systems work well in *A. thaliana*,^95^ rice, wheat, and rapeseed (*Brassica napus*),^96^ but showed lower efficiency in other organisms than its original counterpart, i.e., SpCas9 or SaCas9.^92,97^ Recently, several ABE variants have been developed for adenine base editing in mammalian cells, which may be useful for efficient editing in plants.^98,99^

#### 2.2.2. Prime Editing-Extending the Dimensions of CRISPR/Cas Based GE

Prime editing in plants is a relatively new technique; yet, it has enormous potential for a wide range of plant gene editing applications. The editing system consists of nCas9 (H840A) with reverse transcriptase (RTase) and a prime editing guide RNA (pegRNA) (Figure-3). The process of prime editing allows for at least 12 kinds of base conversions in specific target genes at sites ranging from 3 bp upstream up to 29 downstream of the PAM sequence.^100^ Two binding sites are critical to the recognition of pegRNA, associated with nCas9 (H840A), a spacer sequence by its 5` region, and a prime binding site (PBS) through 3` regions to edit the target DNA sequence. The PBS region acts as a primer for nCas9-linked RTase and the pegRNA is used as a template by RTase; it directly copies the genetic information from pegRNA to be incorporated into the target genome.^100^ After the completion of the reverse transcription, the 5` and 3` unedited flaps equilibrate, trailed by the integration of the modified DNA into the target sequence in the genomic DNA through DNA repair system and ligation reactions.^100^ Recently, prime editing was applied in wheat, rice, maize, and potato.^101^ Consequently, it is pertinent that more reports displaying the wide applicability of prime editing GE system in crop plants are needed to accelerate the progress of this technology in the improvement of crop plants.

## 3. Crispr/cas-Widening the Editing Frame: From Single Gene Editing to Multiplex GE

In living systems, cellular processes are grossly controlled by a wide array of redundant genes. Consequently, mutating a gene may influence the desired phenotypes of an organism, even though in few cases, the mutated gene may be compensated by other genes of a similar family. Hence, it is pertinent to upgrade the functional diversity of GE systems to multiplex gene-editing systems for widening its applications. Against this motive, scientists have developed a CRISPR/Cas9-mediated multiplex GE system where several sgRNA cassettes are controlled by a single of multiple promoters loaded in a single vector.^43,102,103^ Furthermore, efficient stacking of multiple traits in elite plant germplasm has been made achievable by multiplex genome editing, and this has a substantial impact on enhancing the complex agronomic attributes. For instance, two sgRNAs were designed to target two homologous of magnesium-chelatase subunit I (CHLI) that have a critical function in the mechanism of photosynthesis and subsequently, the disruption in both the genes resulted in albino phenotypes in *Arabidopsis*.^32^ The application of multiplex GE was also validated through the disruption of four subunits of katanin p80 by using three sgRNAs, producing a dwarf phenotype of *A. thaliana*.^104^ Tang and colleagues used a multiplexing system based on multiple sgRNAs with Cas9 proteins encoded as a single transcript for GE of various genes in plants.^105^ Besides, for carrying multiplex GE, the CRISPR-Cpf1 system was widely used in crop plants.^106^ Li et al. displayed the application of multiplex gene editing through the application of CRISPR/Cas9 by concurrent editing of 08 target sites in wheat genome.^103^ For instance, the construct consisted of a single promotor controlling numerous repeated units of crRNA, recognized by Cpf1, to produce cleavage at the specific target sites.^106^ These reports strongly suggest that multiplex gene editing by CRISPR systems are expedient tools for targeting multiple genes at once to reduce time cost in knowing the functions of the desired genes/gene families.

## 4. Generating High-Throughput Plant Mutant Libraries Using CRISPR Platform

Another way that CRISPR technology dominates the current trends of genomic research is by developing genome-wide high-speed screening approaches that can thoroughly analyze the influence of regulatory sequences or specific genes on the phenotype of interest.^107^ Traditional plant mutant libraries relying on random mutations and generated through irradiation, ethyl methane sulfonate (EMS) mutagenesis, T-DNA insertions, and transposons require many generations to express loss-of-function mutants. CRISPR/Cas-based whole-genome-scale expression libraries can be constructed, according to computation, for extensive functional genomics and genetic improvement. By delivering the gRNA library, together with Cas9, into cells would generate a cell population to knock out each gene in the genome.^108^ Recently, two independent research groups designed large scale CRISPR/Cas9-based knockout mutant libraries that covered most of the genes in rice plants.^109,110^ Lu and colleagues generated 90,000 transgenic rice lines by targeting 34,234 genes, while a single generation of homozygous mutants was obtained.^109^ Similarly, 13000 genes expressed in rice shoot tissue were targeted, resulting in 14,000 independent T0 lines.^110,111^ Recently, Jacobs and colleagues constructed mutant expression libraries of leucine-rich repeat genes of subfamily ΧΙΙ (immunity-associated genes) in the tomato plant.^111^

Recently, Liu, Jian^112^ used integrated CRISPR/Cas9-based high-throughput, targeted mutagenesis to target 743 candidate genes in maize, governing agronomic and nutritional traits.^112^ It has been demonstrated that incorporating forward and reverse genetics through a focused mutagenesis library ensures that the essential agronomic genes with complex genomes are easily validated. They first designed an improved high-throughput GE platform in maize to target >1,000 maize candidate genes, potentially governing different agronomic traits, which could be applicable to other species with genomic complexity. Furthermore, to validate the phenotypes associated with genetic traits, this targeted mutagenesis workflow can be used as an unprecedented tool for dissecting classical genomic intervals and identifying novel genes, phenotypic modifications, and addressing the widely recognized gene redundancy issue in identifying gene function in plants. The knowledge-based targeted mutagenesis is certainly an effective and high-throughput approach to functional gene identification. This method can be exploited in other crops as well. Nevertheless, large-scale screens with a sgRNA library are eventually accessible to the plant community. Future advances in the high-throughput phenotyping approach, together with the emerging techniques, offering high transformation efficiency for a large array of crop species and improved sgRNA delivery efficiency by new DNA carriers without tissue culture would be critical for further large-scale exploration of mutants and precise crop breeding.^113^ To enhance the utility and robustness of CRISPR-based screens, various scientific groups have refined and optimized screening approaches as well as developed computational tools that could help in designing large-scale CRISPR-based screens, including CRISPRa (CRISPR activation) and CRISPRi (CRISPR inhibition) tools.^114–116^

## 5. Integrating Cis-Genesis and CRISPR/Cas Systems: Harnessing Precise Crop Breeding

Previously, CRISPR/Cas was used to target coding regions to generate null alleles. While this application notably enables heritable alleles for reverse genetic analysis, selection of loss-of- function mutations in coding sequences may culminate in deleterious or pleiotropic effects.^117,118^ On the other hand, gene expression modulation can use cis-engineering to be advantageous for crop improvement, with fewer detrimental pleiotropic effects.^119,120^ The key techniques for editing a gene or a genome are genetic mutations and transgene generation. Somatic hybridization enables the genetic material fusion between the two species separated by genetic barriers. Recent advances in sequencing platforms have led to the isolation of cis genes (genes from crossable species). The process of cis genesis transfers the entire gene, including its regulatory sequences in sense orientation, to sexually compatible species or a closely related species.^121,122^ In contrast, intragenesis refers to a process when coding and regulatory sequences of a gene are assembled either in antisense orientation of in sense orientation.^123^ In theory, unlike intragenic, cis-genics can be accomplished by conventional breeding. The acceptance of cis-genic plants is more since genes are inserted from the crossable species, and the process is similar to conventional breeding. Nevertheless, in both cases, only the genes coding for traits to be improved should be ultimately traced in the regenerated crop plants, and the use of selectable marker genes can be avoided using various alternative technologies, such as cigenesis, steroid-inducible recombinase platform.^122,124^

Besides, cis-engineering through CRISPR/Cas-mediated have also succeeded in the epigenome. CRISPR/Cas9 has been used to modify the epigenome of an organism for switching OFF/ON specific gene modules. For instance, the dead Cas9 (dCas9), a catalytically inactive Cas9, has been fused to inhibitors and activators of transcription to target the specific promoters or enzymes/proteins that modify the chromatin to edit the epigenome of the target organism.^125,126^ However, only a few reports have been defined in *Arabidopsis* that show epiGE by DNA methylation modifications^127^ and histone acetylation.^128^ Until now, there are only a few case studies under epiGE that focused attention on the applications of cis-engineering of DNA. Moreover, various recent articles have reviewed the application of cis-genic approach in enhancing the tolerance to diseases and improving good quality traits in different crops such as apple, grape, potato, poplar, and durum wheat.^122–124^ Vanblaere et al.^129^ reported the molecular characterization of cis-genic apple (Gala) lines with Rvi6 scab resistance genes, whereas, Würdig et al.^130^ used cis-genesis based on the Flp/FRT recombinase system to develop apple cultivars with the same trait. Besides, Jo et al.^131^ developed marker-free potato lines that are resistant to late blight from *S. stoloniferum* (Rpi-sto 1) and *S. venturii* (Rpi-vnt1.1) through *Agrobacterium*-mediated transformation by the cisgenic approach. Since the gene sequences transferred through cis-genesis are acquired from the same or closely related species, the information of their gene position, sequence, and function in the genomes is crucial. This information will offer timely access to a wider application of cis-genic technology instead of transgenesis. Rodríguez-Leal et al.^119^ demonstrated that CRISPR/Cas9 engineering of promoters creates cis-regulatory alleles to enable quantitative variation for breeding of crops. This group edited promoters of genes that regulated three important characters in tomatoes (*Solanum lycopersicum*), i.e., inflorescence branching, fruit size, and plant architecture. Results showed that this method enabled rapid selection of unique alleles and fine manipulation of yield components in crop plants. Besides enhancing variation for different traits, this study provides a basis for analyzing complex associations between gene-regulatory alterations and quantitative traits. Furthermore, the cis-genic approach reduces both linkage drag and duration of selection, and the modification of genotype and phenotype characters remained largely unchanged. Remarkably, CRISPR/Cas9-driven sanitized genetic screens enable recovery of a pool of cis-regulatory alleles with a wide range of phenotypic effects, providing scope for increasing the genetic diversity in crops.^119^

Data reveals CRISPR/Cas system is capable of targeting functional cis-regulatory elements for enhancing genetic variations in crop plants.^132^ Until now, a few studies have been reported regarding CRE deletion or disruption to effectively control the target gene expression in crop plants. Duan, et al.^133^ studied the expression patterns of all five *OsRAVs* under salt stress. Out of them, only *RAV2* gene was transcriptionally controlled under enhanced salinity stress. CRISPR/Cas-mediated cis-engineering is used to mediate precise mutations in the GT-1 element of the *OsRAV2* promoter. The results strongly specify that the GT-1 element switches the salt response of this gene. In another study, the promoter of barley (*Hordeum vulgare*) phytase gene, *HvPAPhya*, was targeted for three CREs, viz. Skn1, GCN4, and RY by CRISPR/Cas9 and TALENs. The plants with mutations at the targeted region indicated a considerable decline in phytase activity, showing the significance of the CREs for gene expression. Li et al.^134^ used CRISPR/Cas9 editing to develop transgene-free bacterial blight resistance in rice by causing deletion in the promoter sequence of xa13. The Xa13 gene in rice normally controls bacterial blight disease resistance and anther development. Thus, this deletion strategy in rice improved disease resistance without affecting its fertility.

Presently, there are a few research articles published on the two-target site-directed deletion and the selection of editing sites in noncoding promoter regions. These gene-editing systems do not mutate coding regions to change their gene expression patterns. Instead, they simply edit the promoter regions that can be central to future genome engineering and breeding.^135,136^ Oliva R, et al.,^137^ used CRISPR/Cas9 to edit TALE-binding elements (EBEs) of three *SWEET* gene promoters in rice, resulting in broad-spectrum bacterial blight resistant lines. Moreover, Xu et al.^138^ employed the CRISPR/Cas9-based editing to engineer broad-spectrum resistance against rice bacterial blight by disrupting the EBEs of *OsSWEET11* and *OsSWEET14 S*-genes. Three state-of-the-art studies in Duncan grapefruit (*Citrus paradise* Macf.) and Wanjincheng orange (*Citrus sinensis* Osbeck) validated the role of CRISPR/Cas editing of the PthA4 effector binding CREs (cis-regulatory elements) in the LATERAL ORGAN BOUNDARIES 1 (*LOB1*) promoter in alleviating canker resistance.^136,139–141^

The CRISPR/Cas-enabled cis-engineering was further exploited to alter known CREs in introns and downstream gene sequences.^142^ YUCCA3 (YUC3) intron containing the CTCTGYTY motif was figured out in Arabidopsis using the chromatin immunoprecipitation sequencing (CHIP-seq) technique. It was found to be involved in engaging RELATIVE OF EARLY FLOWERING 6 (REF6) to its target site.^19^ Removing four repeats of the CTCTGYTY motif blocked REF6 binding at the mutant loci. Also, CRE of 450 bp present in the 2nd intron of Arabidopsis AGAMOUS (AG) was excised with CRISPR/Cas9 to confirm its role as an activator of the AG gene expression. The excision of this CRE leads to early flowering after a 40% decline in its expression.^143^ Promoter swapping and insertion is accomplished via HDR with unprecedented potential to improve different crop traits. However, the low efficiency of HDR in higher plants has been challenging.^144^ Till date, only three cases have been investigated, wherein promoters were precisely introduced or swapped through CRISPR/Cas9-mediated HDR.^145–147^ The 35S promoter was introduced to the upstream of the anthocyanin 1 (ANT1) gene, controlling its biosynthesis and enhancing the accumulation of anthocyanin tomato tissues.^145^ In another study, HDR was exploited to insert and swap the GOS2 promoter in the 5′- UTR of ARGOS8 gene in maize. The mutated plants exhibited enhanced expression of ARGOS8 and improved grain yield in drought stress conditions.^146^ Moreover, cassava (*Manihot esculenta*) resistance to glyphosate was created by a promoter swap of the *ESPS* gene.^147^ These promising results reveal the prospect of harnessing CRISPR/Cas-enabled cis-engineering to improve various traits, for example, crop yield, quality, and stress tolerance. Besides, there is higher knowledge of transferred sequences in cis-genesis compared to conventional breeding.^123^ Moreover, numerous reports confirmed a larger consumer acceptance of cis genic-derived products, unlike the corresponding transgenic ones.^123,124,148^

## 6. Power of CRISPR/Cas for Enhancing Hybrid Breeding

Various recent studies have established the prospects of CRISPR to generate high-quality hybrid varieties, which are mostly based on the male-sterile maternal lines.^15,149^ A remarkable effort was made in generating male-sterile lines using CRISPR/Cas-mediated gene knockouts, including photosensitive male-sterile in CSA rice,^150,151^ ms45 wheat lines,^152^ and thermosensitive male-sterile tms5 rice lines.^153^ Shen, Que^154^ demonstrated that male fertility could be rescued in *Japonica indica* hybrids by knocking out *1–2 Sc* gene copies in the indica allele Sc-Ι. In the same way, Yu, Miao^155^ observed improved fertility in japonica-indica hybrids by knocking out the toxin gene ORF2, which is responsible for the felo-de-se of selfish-gene in rice. In another study, two genes, *SaF/SaM* and *OgTPRI*, at the sterility locus, Sa and S1, respectively, were disrupted to conquer the reproductive barriers in *Japonica indica* hybrids.^156,157^ Recently, GE was used to knockout three genes, *REC8, PAIR1*, and *OSD1*, involved in rice meiosis to substitute mitosis.^117^ Asexual propagation lines were developed by two independent groups, either by knocking out the *MTL* gene^158^ or by the activation of *BBM1* in egg cells,allowing to fix hybrid heterozygosity via seed propagation.^159^

## 7. Exploiting the Genetic Diversity of Wild Plants

Out of the more than 300,000 plant species available globally, only up to 200 plants are utilized at the commercial level. Moreover, only rice, maize, and wheat crops are the main energy sources for human consumption. Continuous modification and improvement of modern crops for thousands of years has led to a loss of diversity.^160^ In addition, generating elite cultivars through further improvement by classical approaches could not always be the most prudent way for producing cultivars with novel traits, resistant to biotic/abiotic stresses. Witnessing the increased sequencing of crop genomes, GE tools offer one the efficient strategies to plant domestication by opening up the vast genetic diversity, from wild or semi-domesticated species, thus generating crops with improved polygenic traits and enhancing genetic diversity in plants. The long domestication process of modern cultivars results in loss of genetic diversity. A wild tomato plants exhibit a higher degree of tolerance to diverse stressors; thus a repository of tolerance genes to accomplish *de novo*-domestication through GE tools. For instance, accelerated *de novo* domestication of wild tomato using CRISPR/Cas9-mediated multiplex editing generated loss of function in four sets of genes coding for flowering time (*SP5G*; *SELF PRUNING 5 G*), plant architecture (*SP; SELF PRUNING*), fruit size (*Sl CLV3; CLAVATA3*, and *Sl WUS; WUSCHEL*).^161^ The gene regulatory elements were targeted to create weak transcriptional alleles and target coding sequences. Moreover, CRISPR-edited plants displayed enlarged fruit size as well as synchronized flowering, determinating the plant architecture without losing tolerance to stresses.

Zsögön, Čermák^162^ reported that *de novo* domestication was achieved in the ancestral tomato, *Solanum pimpinellifolium*, using the multiplex CRISPR/Cas9 approach, resulting in functional disruption of 06 domesticated genes coding for growth habit, nutritional traits, and yield components. They observed a ten-fold increase in fruit yield and a three-fold increase in fruit size within a single generation as compared to the wild plant. Moreover, fruit shape was found to be better and the nutritional quality increased by two-fold in lycopene content, which is equal to five-fold increase in translation compared to modern tomato cultivars. Rapid *de novo* domestication using the CRISPR/Cas approach was applied in an underutilized crop of Solanaceae family, ground cherry (*Physalis pruinosa*). A remarkable success was achieved by considering the efficient transformation procedure, gene annotation data, and reference genome. In turn, plants were produced with higher yields and increased fruit size.^163^ In comparison to many other oilseeds, weed pennycress (*Thlaspi arvense* L., Brassicaceae) has a short life cycle, tolerance to extreme cold stress, and high seed oil content. It has homology with other advanced members of Brassicaceae.^164^ Genes controlling seed dormancy (*DOG1*), oil content (*DGAT* genes), accumulation of glucosinolates (*HAG1* and *GTR2*), and oil quality (*FAE1* and *FAE2*) can be modified via the genome-editing technologies that should significantly contribute to the development of elite pennycress varieties. In addition, progenitors of teosinte (*Zea mays* spp. parviglumis), wild rice (*Oryza rufipogon*), and wild emmer wheat (*Triticum dicoccoides*) could be undertaken for re-domestication to promote agricultural diversity and decode a lot of problems facing sustainable agriculture.^117^

Recently, Ivanizs, Monostori^165^ investigated the genetic diversity of *Aegilops biuncialis*, one of the treasured sources of agronomically important traits, which may considerably enable the introgression breeding of wheat. A correlation of the intraspecific variation in the heading time trait using analysis of variance (ANOVA) and principal component analysis (PCA) divulged four phenotypic groups, presenting association with the genetic structure and geographic distribution, apart from minor differences. The comprehensive study of genetic and phenologic differences provides an understanding of the adaptation of promising genotypes of *A. biuncialis* that could be exploited for wheat improvement. The CRISPR-Cas approach can be used to target agronomically important genes in *A. biuncialis* for wheat improvement. Thus, CRISPR-mediated *de novo* domestication of wild plants offers a new window for crop improvement.^166^ Furthermore, exploiting wild crop relatives in the quest for a repository of allele mining could pave the way for developing germplasm for a wide range of crop varieties, having tolerance to biotic as well as abiotic stress factors.^167^ Consequently, there is a huge scope for the use GE tools such as based on CRISPR/Cas mechanism for *de novo* domestication of neglected, semi-domesticated, and wild crop plants for food and national security.

## 8. A Combinatorial Approach to the Development of Disease-Resistant Crops

**Figure 4.**
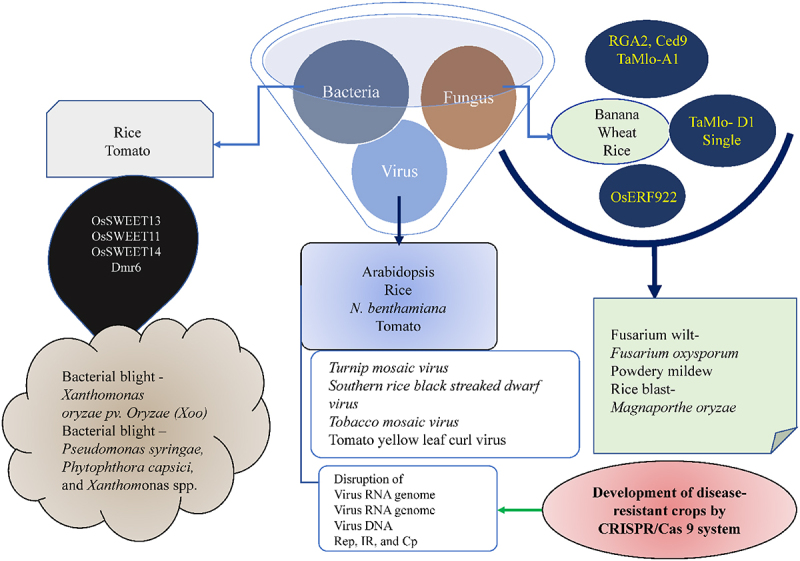
Engineering crops for disease resistance. Schematics showing the examples of genes edited by the CRISPR/Cas system to enhance the resistance to bacteria, fungus, and virus..

Induction of biotic stress to crop plants occurs majorly through virus, bacteria, fungi, nematodes, and insects. In addition, the constant increase in the number of new strains of microbial strains further complicates the battle against pathogens.^168,169^ Therefore, it is pertinent to unravel the plant interaction to protect agricultural crops from these deadly biotic stressors.^170^ GE technologies such as CRISPR/Cas9 have been successfully used to understand the response of plants to pathogens and investigate the molecular basis of plant-pathogen interactions to develop disease-resistant crops (Figure 4, Table 1).^171,172^ For instance, disease-causing genes such as “S-genes” have been disrupted by using GE based on CRISPR/Cas9 systems to develop disease resistant crops. Similarly, resistance to rice blast and reduced blast lesions caused by *Magnaporthe oryzae* by the knockout of ethylene-responsive gene *OsERF922* in plants were observed by using the CRISPR/Cas9 GE tool.^173^ Likewise, targeted mutagenesis of *SWEET13* gene produced bacterial blight-resistant plants upon application of CRISPR/Cas9 GE.^174^ The improved resistance against *Xanthomonas citri* was due to the frame-shift mutation and disruption of the *CsLOB1* gene.^175^ Shan Q, et al.^176^ applied the CRISPR/Cas9 technique in wheat protoplasts for editing the *TaMLO* gene^177^, producing wheat lines resistant to powdery mildew due to infection of caused by *Blumeria graminis* f. sp. *Tritici*.^178^ In a further study, resistance to powdery mildew was developed by mutating 03 homologs of the *EDR1* gene by using multiplex GE by CRISPR/Cas9.^179^ Similar results were obtained in tomato by generating mutation in the *MLO* gene using CRISPR/Cas9 to develop resistance against powdery mildew.^180^ For enhancing virus resistance, sgRNAs and Cas9 were targeted in plant genomes for overexpression to inhibit the Gemini virus infection.^181,182^ To tackle wide range of viral diseases, CRISPR/Cas9 system has been widely applied to mutate the target viral genomes.^183^ *Francisella novicida* (FnCas9) is a new discovered ortholog of Cas9 employed for the efficient editing of RNA virus genomes. This ortholog, FnCas9, has been successfully applied in tobacco mosaic virus and cucumber mosaic virus, inhibiting the replication and providing immunity against them.^184^ Consequently, GE through CRISPR/Cas9 toolbox is a highly efficient and exceptional tool to improve the genetic makeup and to combat various pathogens.

**Table 1.** Role of CRISPR/Cas9 in the development of disease-resistant crops.

Crop	Pathogen	Target Gene	Trait Improvement	Reference
Oryza sativa	Rice tungro spherical virus	*eIF4G*	Resistance to rice tungro spherical virus	^246^
Vitis vinifera	Botrytis cinerea	*VvWRKY52*	Resistance to botrytis cinerea	^247^
Solanum lycopersicum	Pseudomonas ssyringae	*SlJAZ2*	Bacterial speck-resistant	^248^
Triticum aestivum	Erysiphe cichoracearum	*EDR1*	Improved resistant to powdery mildew	^179^
Oryza sativa	X. oryzae, pv. oryzae	*OsSWEET13*	Resistance to bacterial blight	^174^
Citrus paradise	Xanthomonas citri subsp. citri	*CsLOB1*	Citrus canker-resistant	^136^
Gossypium hirsutum	Verticillium dahliae	*Gh14–3-3d*	Resistance to cotton verticillium wilt	^249^
Oryza sativa	Magnaporthe oryzae	OsERF922	Resistance to blast fungus	^173^
Solanum lycopersicum	Oidium neolycopersici	*SlMlo1*	Improved resistance to powdery mildew	^250^
Cucumis sativus	Multiple viruses	eIF4E	Broad virus-resistant	^251^
Citrus sinensis	Xanthomonas citri subsp. citri	*CsLOB1*	Citrus canker-resistant	^175^

## 9. CRISPR/Cas9 to Produce Climate-Smart Crops

In order to cope with various abiotic stressors, the CRISPR/Cas9 technology is widely applied in major crop plants such as, rice, maize, wheat, tomato, cotton, soybean, and potato. Moreover, CRISPR/Cas9 tool has revolutionized the modernized plant-breeding strategies due to its critical role in producing climate-smart crops, resilient to a wide range of abiotic stressors.^15,185–191^ For instance, the functional studies of *TaDREB3* and *TaDREB2* genes located in wheat protoplast have been investigated by the CRISPR/Cas9-based GE.^186^ Further, with a T7 endonuclease assay, approximately 70% of the transfected protoplasts have been confirmed to express mutated genes.^186^ Similarly, in rice plants, the 03 genes, *viz*., phytoene desaturase (*OsPDS*), betaine aldehyde dehydrogenase (*OsBADH2*), and mitogen-activated protein kinase (*OsMPK2*) are edited by employing CRISPR/Cas9 GE tool. The transformation of CRISPR/Cas9 against the genes responsible for mediating stress tolerance is done by protoplast transformation and particle bombardment.^176^ Plant annexins, including *TdANN12*, *StANN1*, *BjANN1,* and *AnnAt8* play a major role in protecting plants from abiotic stressors. In addition, CRISPR/Cas9 system was employed to knockout the annexin gene *OsAnn3* (a calcium-dependent lipid binding annexin) responsible for cold stress in rice.^154^ Similarly, base editing system was used to validate the functions of *SAPK2* gene using its critical role in salinity and drought stress.^192^ In conclusion, CRISPR/Cas systems speed up the production of climate-resilient crop plants in the scenario of global climatic changes.

## 10. Organelle GE Through CRISPR/Cas System: An Overview

GE tools have emerged as the most dominant technique to develop important crops that have widespread applications in agriculture. Almost all research findings pertaining to GE of plant genomes are undoubtedly focused on plant nuclear genes. Scientists are still finding ways to modify the plastid and mitochondrial genomes since both plastome and mitome are vital for plants because of their much smaller size and because they contain nonredundant genes and genes for metabolic pathways.^193^ Regardless of the progress in the editing of nuclear genomes, there is an important set of genes present in chloroplasts and mitochondria that plays key roles in photosynthesis, respiration, and development.^193,194^ These extra-nuclear nonredundant genes and metabolic pathways genes could be targeted for enhancing agricultural productivity.^195^

Engineered nucleases are used to induce double-stranded DNA breaks in mitochondria.^196,197^ However, researchers need to introduce DNA templates into organelle genomes to repair and introduce desirable modifications at the break sites. For this purpose, methods and protocols are obligatory to isolate the stable organelle transformants. In the case of eukaryotes, these protocols are only available for chloroplasts, and the success rate in plastome engineering depends on the efficiency with which plastome transformation and regeneration of transplastomic plants is achieved. The engineering of the photosynthetic pathways for carbon metabolism and nitrogen fixation are two major challenges that plastome engineering can address. The key chloroplast-editing target gene, *rbcL*, encodes for the large catalytic subunit of Ribulose bisphosphate carboxylase/oxygenase, essential for carbon dioxide fixation, and is the focus for improvement.^198^ Photosynthetic ability to reengineer genomes involves introducing and coordinating 20 genes or more to provide C_4_ photosynthesis in C_3_ plants. Photosynthetic efficiency can be enhanced by improving light capture, light energy conversion, CO_2_ uptake, and a prominent canopy.^195^ Due to the variations in the nuclear and plastid genome characteristics, different engineering approaches came into existence.

The eukaryotic nuclear genome has the architecture, regulation, and processing of the eukaryotic genes. DNA insertion into the plant nuclear genome is most often achieved via random *Agrobacterium*-mediated transformation whereas plastome is an alternative compact option for targeted editing. Moreover, each gene has its own promoter, i.e., monocistronic gene structure is a general rule. On the contrary, the organelle genome is prokaryotic, and its genes are derived from operons with untranslated regions that play a pivotal role in gene expression. Foreign DNA is usually bombarded into plastome and mitochondria using a gene gun and is then incorporated at the target regions through homologous recombination. The type of genome to be edited relies on the choice of research or product. For example, single-gene governed traits that are achieved through either knockin or knockout are better targets for nuclear engineering, but plastomes and mitomes are better suited for manipulating metabolic pathways. Recently, it was observed that few noncoding RNA might possibly facilitate the import of foreign mRNA into plant chloroplasts.^199^ Such RNA and protein delivery tools could open up the leeway of plant cell organelles’ DNA-free GE by unswervingly importing mRNA, protein, or CRISPR ribonucleoprotein complexes. The field of editing the plastome and mitome is a completely open challenge to scientists for modifying crop plants for food and nutritional security.

## 11. Biosafety Concerns of Gene-Edited Plants

The beginning of the editing of genomes generated not only enthusiasm but also debate, throwing up global regulatory and governance challenges, since it is evident that the use of physical as well as chemical mutagens involved in traditional plant breeding usually generates a large number of mutations in plants that are not desirable for the improvement of crops.^200^ However, off target effects based on plant GE may not cause genetic complexities in plant breeding as the undesirable mutations are eliminated by backcrossing. CRISPR-Cas9 has been a flexible and robust tool for gene regulation and genome-editing in eukaryotic organisms, including plants.^32,201^ However, over time, it became apparent that the CRISPR-Cas9 system is affected by diverse factors such as sgRNA design, delivery methods, target site selection, Cas9 activity, off target effects, and the frequency rate of HDR.^32,202–204^ The most important apprehension is the vulnerability of producing undesirable gene alterations in plants, owing to the off target activity.^202–204^ Furthermore, simultaneous gene editing by multiplexing will further create serious regulatory concerns, largely due to possibility of gene rearrangements.^205^ The major concern to the CRISPR/Cas9 GE is off-target mutations that will have deleterious effects on thearget genome. Consequently, precision modifications by using CRISPR/Cas9 GE and its risk needs to be studied.^23,38,206^ Off-target mutations can also threaten the environmental safety and integrity. This may happen by the transfer of off target mutations to other organisms. For instance, it is observed that, over time, the efficiency of GE in soybean embryos is enhanced by using the CRISPR/Cas9 system.

Gene drive is another worrying element as far as crop safety disquiets of GE technologies. The process of gene drive involves biased inheritance of genes from donor parents to the offspring.^207^ The naturally existing gene drive is held in check by the naturally inbuilt mechanisms of plants.^207^ It is reported that the CRISPR/Cas9 system may create highly efficient gene-driving genetic elements, making sure that researchers understand the clear containment and confinement conditions of the gene-drive possibilities. All the sam, we believe that there is limited possibility of gene drive in domesticated plants due to controlled breeding and a longer generation time.

Interestingly, DNA-free GE with *in vitro* preassembled CRISPR/Cas9 ribonucleoproteins (RNPs) prevented the off target mutations and transgene integration to unpredictable levels, thus improving the CRISPR-Cas9 specificity in plants.^42,202,204^ However, the presence of cell wall makes it a challenging task for transformation through electroporation, lipofection, and microinjection in intact plant cells. However, in recent times, this problem has been solved by inserting Cas9 RNPs into protoplasts of lettuce, followed by tissue regeneration.^208^ Moreover, Svitashev, Schwartz^209^ used biolistic bombardment successfully to deliver Cas9 RNPs or *in vitro* transcripts (IVTs) into young embryos of wheat and maize. Hitherto, these transgene-free approaches are possible only with few plant varieties and species due to the high cost, less stability, and high technological inputs.^204,210–212^ Various research groups have done substantial work to decrease the off target activity of Cas9, together with improving gRNA design, delivery of RNPs, and protein engineering by systematically controlled Cas9 and gRNAs via an excess of environmental or chemicals or by employing artificial genetic circuits, which control the CRISPR function.^202,204,209^ Similarly, improvements in base editing to enhance its specificity are achieved by restricting the deaminase activity outside Cas9 binding using various engineered deaminases or by using deaminase effectors to reduce its affinity to bind DNA.^91,213^

CRISPR/Cas9 technology also faces concerns linked to the Cas9 protein because it was revealed that an immune response was induced in mice through adeno-associated viral delivery of Cas9.^204,214^ Besides, Cas9 specificity and the limited number of targeted sites, owing to the PAM requirement, is a matter of concern.^215,216^ Moreover, functional characterization of gRNAs, prior to application, is significantly important because of the varying gRNA activities, thus making gRNA selection difficult.^216^ In addition, the intervention of engineering proteins for establishing the feasibility of engineering Cas9 that changes PAM specificity, improves its fidelity, and identifies other important motifs, which are of great importance.^204,217,218^ Moreover, the alterations to gRNA and Cas9 design, such as dimeric Cas9-*FokI* fusions, paired Cas9 nickases, and utilization of truncated sgRNAs (GN17-NGG or GN18-NGG), have significantly enhanced the on targeting activity of CRISPR/Cas nucleases.^219,220^ Additionally, Cas9 variants, other homologs from bacteria, or six novel programmable CRISPR-Cas nucleases of Class 2 system were uncovered in bacterial genomes using functional and computational analyses.^203,221^

## 12. The Regulatory Landscape of Genome-Edited Crops

Plants, whose genome was altered in a unique way from natural processes (mating and/or natural recombination), are referred to as genetically modified (GM).^222,223^ On the other hand, GE refers to DNA modifications that can hardly be distinguished from equal modifications achieved through conventional plant breeding or natural methods.^201,204,224^ The regulation of genome-edited plants was comprehensively debated by policymakers, scientists, and regulatory authorities.^225–227^ However, different countries have followed different patterns in generating, consuming, and regulating GM plants. At the same time, several countries ban their production and avoid consumption, whereas others adore the consumption and production of GM plants.^204^ Owing to the escalating disagreement between scientific advancement and legal regulation, the German Research Foundation and Science Academies concludes that the principally process-based European regulatory method is not permissible. Consequently, it is not justifiable that budding risks can only stem from the organisms as a product of the breeding and not from the process itself.^228^ Therefore, the report proposes, as a first and foremost step, to revise the European GE regulation in the short term. “In a second and long-term step, the legal agenda should be renovated not on the fundamental process but on the novel traits and characteristics of an organism that are related to the health, environment and nature conservation.^224,229^

However, genome-edited plants or animals are being regulated in some countries based on biosafety frameworks developed through specific legislation, even though some countries do not consider GE organisms as GMOs.^204,230^ Hurdles in the regulatory process of plant GE comprise market access and addressing the public’s concerns about its safety, without restraining the technological advances.^230,231^ Consequently, the marketing of gene-edited plants and their resulting products might circumvent the firm biosafety protocols that are mandatory for transgenic plants.^224,232–234^ In March 2018, the United States Department of Agriculture (USDA) stated that GE, in some cases, is the same as the conventional approach and thus does not involve any regulatory error in the American Regulatory context.^224,235^ The first CRISPR-edited crops commercialized in the USA without any regulatory protocols. These include mushrooms resistant to browning and a waxy corn composed entirely of amylopectin.^204,235^ The judgment about not to regulate established on the statement that no transgene was introduced during editing, and the subsequent modification did not encompass any pesticide or herbicide resistance.

In contrast, Canada has been faithful to the scientific values placed in its domestic regulatory modus operandi for GM plants, developed 25 years back. According to the Canadian policy, any genome-editing tool that produces a product having novelty is subjected to further regulatory checks related to allergic reactions and impacts on nontarget organisms.^236,237^ The first gene-edited products that are approved in Canada are potatoes with nondark spots and nonbrowning apples. Consent was provided after a prolonged assessment procedure that showed that gene-edited apples and potatoes did not cause ill effects to human health as compared to currently available apples and potatoes in the Canadian market.^235^ Furthermore, Argentina has established a functional regulatory protocol for certifying genome-edited products.^204,238^ Regulatory bodies and policy makers have developed assessment protocols that are related to the Cartagena Protocol on Biosafety, depending on appraisals.^204,239^ If a product developed from a transgenic technology is DNA (transgene)-free, then this product can be categorized as non-transgenic. The almost same protocol is followed by Chile and Brazil. Both countries regulate gene-edited plants on a case-by-case evaluation and exclude them from biosafety protocols when the final product is without transgene. For now, European Union (EU) countries stay politically conflicted about GM crops.^235^ Recently, the Court of Justice of the European Union (ECJ) ruled that gene-edited crops should be subjected to similar strict regulatory guidelines as GM organisms.^204,239^ The ECJ in its judgment stated that only mutagenesis techniques that are usually used in plant breeding and have an extended safety record are exempted from this regulation. Gene Technology Act (GT Act) was introduced in 2000 in Australia, specifying that a GMO is an organism that is created by altering its genes or genome. The Gene Technology Regulations were launched in 2001, in which schedule 1 postulates that organisms are generating from the genetic material exchange. Further, an amendment was made in October 2019 to schedule 1 that eliminated GMOs improved by CRISPR-Cas9 GE tools.^230^

In 2016, the Hazardous Substances and New Organisms Act 1996 (HSNO Act) Act was amended in New Zealand in an article explaining that GE-based plant breeding is subject to the same regulations as applied to GMOs.^91^ In India, the process of regulatory framework was established in 1989 for the purpose of generation and utilization of GMOs and their respective products through novel genetic tools. The Food Safety and Standards Authority of India (FSSAI) defined modified food or genetically engineered as “any food or food ingredient containing GM or engineered organisms obtained through gene technology, or food and food ingredients produced from but not containing GM or engineered organisms obtained through gene technology.” Thus, the regulation of all novel GE tools, including CRISPR-Cas9 GE technology, fall within the regulatory protocols.^240^

Japan’s Ministry of Health, Labor, and Welfare (MHLW) has released a regulatory policy on March 27, 2019, for the handling of food products derived from GE technology. The policy established that genome-edited transgene-free foods are not classified as GMOs and are not subjected to regulatory assessments. The provision states that the GE technology based on single or few nucleotides can occur naturally. Such changes are difficult to differentiate from mutational changes that occur in traditional breeding techniques. The new MHLW’s policy also specifies that off target mutations in genome-edited foods are not a matter of concern as such mutations are also detected in crops produced by conventional breeding.^204,241,242^

It appears that the choice to regulate or deregulate GM crops and foods by different countries is determined based on the type of existing GMO regulations. Those countries that have implemented a process-based regulatory protocol consider only those products that are developed by the regulated process. These products are primarily different and riskier than similar products produced by other methods that likely regulate foods, and GE crop plants coming under the GMO acts. In contrast, countries that follow a product-based regulatory protocol and regulate GE based on the final product characteristics instead of the process by which it was developed might not regulate foods, and GE crops under GMO rules. Countries such as, for example, Malaysia and Thailand, adopted both product- and process-based approaches, and they also possibly regulate foods and GE crops under the regulation of GMO laws.^224,240^

## 13. Conclusion and Future Directions

Over the last decade, we have seen a period of dramatic change in the rapidly developing GE technologies at the forefront of the effort to evolve crop plants for desirable traits. Large-scale production of low-cost, safe, and nutritious food by adopting a sustainable agricultural system is a major challenge for agricultural scientists in the current scenario. This goal can be achieved by using modern technologies to improve wild crops. Among these, genome-editing technologies are at the forefront to modify crop genomes to enhance production and create resilient features. The GE technologies employed for decades including ZFN, TALENS, Cre-LoX-P, and CRISPR/Cas system have been used for crop improvement to settle the issue of food security and nutritional quality.^243,244^ Many barriers to genome editing have been addressed by the use of CRISPR/Cas9 toolbox, which has also given crop improvement ideas a new start. In addition, they are integrating cis-genesis and harnessing precise crop breeding. Moreover, the power of CRISPR/Cas is validated by its applications to enhance hybrid breeding and enhance plants’ genetic diversity. The introduction of CRISPR/Cas GE provides scientists with an easy way to modulate target crop plants for a specific class of genes in a more efficient and precise manner. Wide usage and versatility of CRISPR/Cas GE tool is effectively practiced in functional genomic studies and molecular crop breeding programs, with incomparable results in terms of producing large crop varieties with special agronomic traits. Exploring the molecular phenomenon involved in stress biology of crop plants through CRISPR/Cas systems led to the development of superior and elite crop plants resilient to various biotic as well as abiotic stress factors.^120^ Given that CRISPR/Cas systems are among the most important gene editing technology employed globally, the tool still has limitations like off targeting in plant genomes. This issue can be resolved by getting the whole genome sequence of the target plant and by designing sgRNAs that are highly tailored to the target sequences.^245^ Other pitfalls include obstacles in the delivery of CRISPR cargoes and demands for devilment of the novel carriers for efficient transformation. Moreover, the crops developed through CRISPR/Cas systems have no involvement of exogenous DNA, and the editing of genomes is very much in compliance with the biosafety regulations. Due to the widespread use of CRISPR/Cas and its variants in figuring out the molecular mechanism behind the fundamental procedure involved in crop improvement, we can come to inference that it is a promising toolkit to engineer climate-resilient future crop plants. ^252,252^ Despite several challenges, the current pace and utilization of CRISPR/Cas systems in crop improvement validates its expedition to serve humans by attaining food, nutrition and climate security.
